# Aging-Associated Molecular Changes in Human Alveolar Type I Cells

**DOI:** 10.35534/jrbtm.2024.10012

**Published:** 2024-07-22

**Authors:** Xue Liu, Xuexi Zhang, Jiurong Liang, Paul W. Noble, Dianhua Jiang

**Affiliations:** 1 Department of Medicine and Women’s Guild Lung Institute, Cedars-Sinai Medical Center, Los Angeles, CA 90048, USA; 2 Department of Biomedical Sciences, Cedars-Sinai Medical Center, Los Angeles, CA 90048, USA

**Keywords:** Aging, Alveolar Type I Cell, Senescence, Epithelial Cell Identity, Tight Junction, Apoptosis

## Abstract

Human alveolar type I (AT1) cells are specialized epithelial cells that line the alveoli in the lungs where gas exchange occurs. The primary function of AT1 cells is not only to facilitate efficient gas exchange between the air and the blood in the lungs, but also to contribute to the structural integrity of the alveoli to maintain lung function and homeostasis. Aging has notable effects on the structure, function, and regenerative capacity of human AT1 cells. However, our understanding of the molecular mechanisms driving these age-related changes in AT1 cells remains limited. Leveraging a recent single-cell transcriptomics dataset we generated on healthy human lungs, we identified a series of significant molecular alterations in AT1 cells from aged lungs. Notably, the aged AT1 cells exhibited increased cellular senescence and chemokine gene expression, alongside diminished epithelial features such as decreases in cell junctions, endocytosis, and pulmonary matrisome gene expression. Gene set analyses also indicated that aged AT1 cells were resistant to apoptosis, a crucial mechanism for turnover and renewal of AT1 cells, thereby ensuring alveolar integrity and function. Further research on these alterations is imperative to fully elucidate the impact on AT1 cells and is indispensable for developing effective therapies to preserve lung function and promote healthy aging.

## 1. Introduction

Human alveolar type I (AT1) cells are specialized, highly flattened epithelial cells that form a crucial component of the alveolar lining in the lungs [1,2]. These cells cover approximately 95% of the alveolar surface area, creating an extensive, thin barrier that facilitates the efficient exchange of gases-oxygen and carbon dioxide-between the air in the alveoli and the blood in the surrounding capillaries [2]. The unique structure of AT1 cells, characterized by their broad, thin cytoplasmic extensions, minimizes the diffusion distance for gases, thus optimizing respiratory efficiency [3]. Beyond their role in gas exchange, AT1 cells contribute significantly to the structural integrity and stability of the alveolar architecture, helping to maintain the shape and function of the alveoli, and to fluid homeostasis, preventing the accumulation of excess fluid in the alveolar space [1,4,5].

Aging impairs lung function, and significantly impacts the differentiation of AT1 cells from their progenitor cells, alveolar type II (AT2) cells [6,7], resulting an age-related decline in alveolar regeneration. We and others have highlighted a series of significant changes in AT2 cells, along with their niches, within aged human lungs that may compromise their capacity to differentiate into AT1 cells [7–11]. More directly, aging is associated with alterations in the morphology and density of AT1 cells [12,13], leading to a decline in their functional activities. Aging-related changes in the lung microenvironment can further impact AT1 cell function and contribute to age-related lung diseases [12]. These findings suggest a significant influence of aging on human AT1 cells, potentially contributing to structural and functional alterations that compromise their capacity to maintain lung health and function. These changes are associated with a higher susceptibility to respiratory diseases. Understanding the effects of aging on AT1 cells is vital for developing interventions to preserve lung health and function in the aging population.

Here, we revisit the single-cell transcriptomics atlas we recently generated with half a million cells from healthy human lungs covering various ages and isolated AT1 cells for further differential and pathway analyses. Intriguingly, the healthy AT1 cells are heterogeneous and at least two functionally distinct AT1 subpopulations were identified. In line with findings regarding AT2 cells [8], AT1 cells from aged donors exhibited significantly increased cellular senescence and elevated chemokine gene expression. In-depth pathway analysis further unveiled an increased resistance of these cells to apoptosis. More notably, aged AT1 cells showed a dramatic deficiency of epithelial identities, evidenced by the downregulation of general epithelial marker genes, as well as genes associated with AT1 cell junctions, the pulmonary matrisome, and endocytosis. This study provides comprehensive evidence demonstrating the impact of aging on molecular and functional changes in AT1 cells and enhances our understanding of age-related declines in human lung functions. As AT1 cells play a key role in gas exchange and maintaining alveolar structure, aging-induced alterations in these cells can lead to increased susceptibility to respiratory diseases and compromised lung health. By investigating the molecular and cellular mechanisms underlying these changes, we can develop targeted therapies to preserve lung function, improve respiratory health, and enhance the quality of life for the elderly population.

## 2. Materials and Methods

### 2.1. Single Cell Transcriptomics Data Analysis

The data collection and integration for the human lungs of healthy subjects from fourteen individual databases containing 94 healthy subjects and 491,187 cells have been previously described [8]. Briefly, the databases were integrated, batch effects were corrected by canonical correlation analysis (CCA), and major cell types were identified based on the expression of canonical marker genes. For AT1 cell identification, commonly used marker genes including AGER, RTKN2, and BDNF were used. The AT1 cell cluster was isolated and re-clustered, and the contaminating cells were identified and excluded with pure AT1 cells retained. Again, the differential analyses were done with GSE135893 dataset excluded as the transcription of some genes in GSE135893 was far beyond the scale of other datasets [8].

### 2.2. Differentially Expressed (DE) Gene Analysis

Differentially expressed genes in AT1 cells from different clusters or ages (young, middle-aged, and aged subjects) were calculated by “FindAllMarkers” as described previously [14]. For heatmaps, gene average expression in each identity was determined by the command “AverageExpression” and a heatmap showing the relative expression of top genes or a specific set of genes was illustrated. For individual gene expression or gene score, Violin plots were utilized with a dashed line indicating the mean levels and a boxplot displaying the distribution of the continuous variable.

### 2.3. Expression Scores of Gene Sets

The command “AddModuleScore” was used to determine the expression score of a gene set as described previously [8,15]. The Cellular Senescence Score was calculated based on the expression of the genes that were detectable in human AT1 cells selected from the core senescence gene list (503 genes) in CSGene database [8,16,17]. The Epithelial Identity Score was determined using common alveolar epithelial genes, and Keratin and Claudin genes, and the Cell Apoptosis score was identified using 81 protein-coding genes participating in the apoptosis pathway from the KEGG Pathways dataset.

### 2.4. Ingenuity Pathway Analysis (IPA)

IPA analysis on the differentially expressed genes of AT1 cells from different clusters or age stages was performed as described previously [8,18]. The DE gene symbols were loaded into IPA together with the expression fold change “avg_log2FC” and “p_val” as inputs. IPA analysis was conducted based on these variables, and the Ingenuity Canonical Pathways and the “-log(*p*-value)” and “z-score” were exported as outputs. Data visualization was depicted using -log(*p*-value) with or without a z-score.

### 2.5. Gene Set Enrichment Analysis

Gene Set Enrichment Analysis (GSEA) analysis on young and aged AT1 cells was conducted with fgsea (Version 1.18.0), as previously described [8]. The hallmark gene sets, C2 curated gene sets and C5 ontology gene sets were downloaded from GSEA web (https://www.gsea-msigdb.org/gsea/index.jsp). The GSAE pathway analysis was performed on all the above gene sets by the DE genes of the aged AT1 cells. The signaling pathways were ranked based on their Normal Enrichment Scores (NES) and *p*-values (pval). The top pathways were visualized by the plotGsea Table and individual pathways were presented by Enrichment Plot with NES, pval, and padj (adjusted *p*-value).

### 2.6. Statistical Analysis

GraphPad was used to show the exact frequency of AT1 cells in each subpopulation. The comparison of each gene expression or score level was visualized using violin plots, and the Wilcoxon test or Kruskal-Wallis test was employed to compare two or three groups, respectively. A *p*-value was included in each comparison by violin plots and the lowest *p*-value calculated by these tests was *p* < 2.2 × 10^−16^.

## 3. Results

### 3.1. Identification of Functionally Distinct AT1 Subpopulations in Healthy Human Lungs

**Figure 1.**
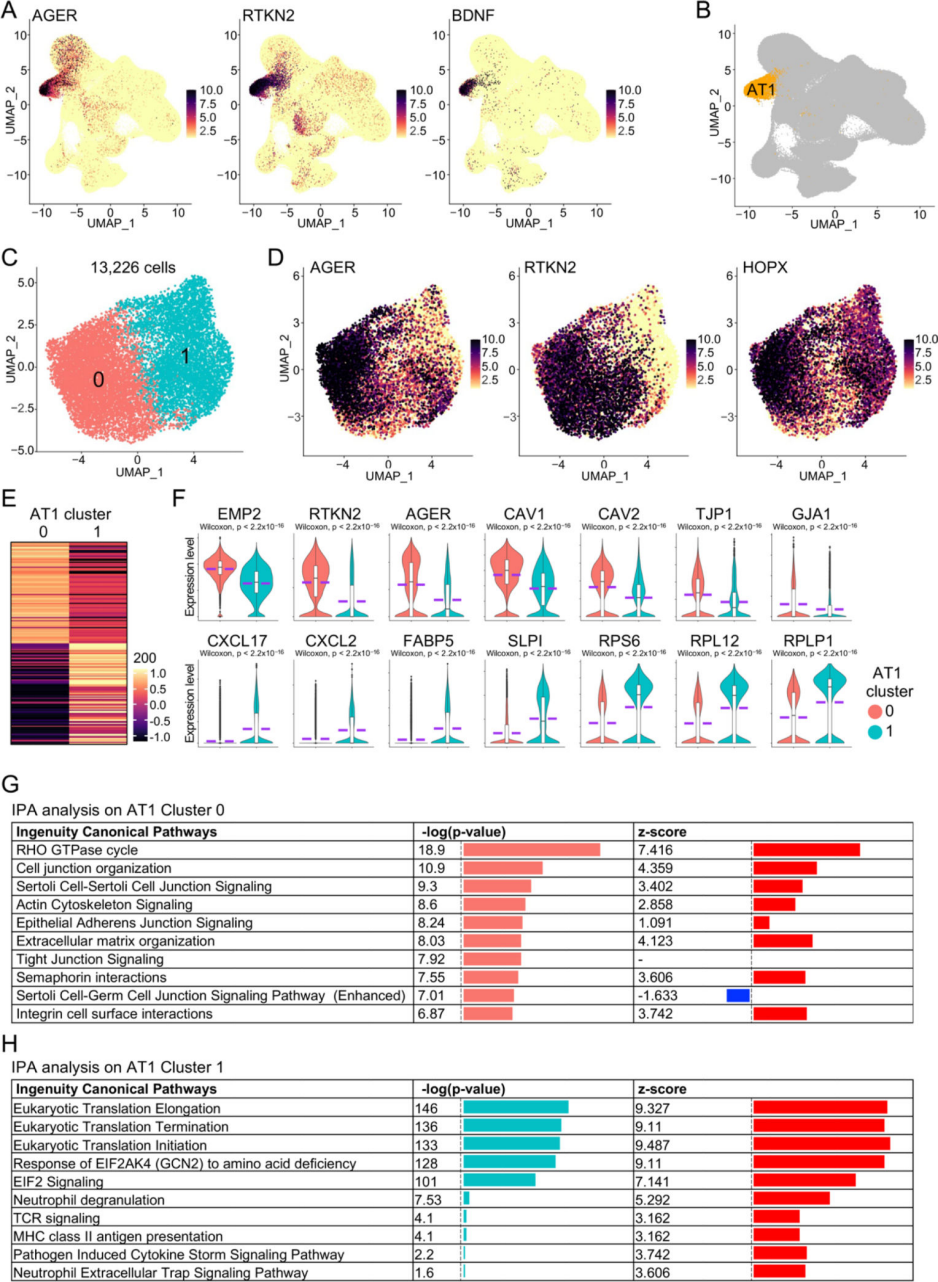
Functional heterogeneity of AT1 cells in healthy human lungs. (**A**) Transcription of AT1 maker genes in the integrated scRNA-seq atlas of healthy human lungs. (**B**) UMAP showed the identification of AT1 cluster. (**C**) AT1 cells were isolated and re-clustered. (**D**) Transcription of canonical AT1 marker genes in the isolated AT1 cells. (**E**) Heatmap displayed the top 100 genes of AT1 cell clusters. (**F**) Expression of representative top genes of AT1 cell clusters were visualized by violin plots. (**G**,**H**) IPA analysis revealed the top Ingenuity Canonical Pathways of AT1 cell clusters.

To systematically study the impact of aging on human lungs, we recently generated a single-cell transcriptomics atlas of healthy human lungs from donors of different age stages [8,17,19–30] and identified an AT1 cell cluster by examining the expression of canonical AT1 marker genes (Figure 1A,B). We then isolated and re-clustered the AT1 subset, removed contaminant cells, and ultimately retained 13,226 purified AT1 cells in two distinct sub-clusters (Figure 1C,D). Differential expression analysis revealed unique gene expression profiles for each AT1 sub-cluster (Figure 1E). Remarkably, the representative top genes in Cluster 0 were predominantly related to the protein components of epithelial cell membranes and cell junctions, whereas those in Cluster 1 were mainly associated with cell inflammation and ribosomal protein genes (Figure 1F). To further verify the specific functions of these two sub-clusters, we conducted IPA analysis on the differentially expressed genes of each sub-cluster. As expected, the top signaling pathways in Cluster 0 were primarily related to epithelial cell junction signaling and organization (Figure 1G). IPA analysis in Cluster 1 identified several top pathways associated with eukaryotic translation attributed to the expression of ribosomal protein genes in this sub-cluster. Additionally, many of the other pathways in Cluster 1 were related to cell inflammation and chemokine storm (Figure 1H), consistent with the elevated expression of chemokine genes in this sub-cluster (Figure 1F). These analyses indicate that AT1 cells in healthy human lungs exhibit significant heterogeneity and at least two distinct sub-clusters are identified, each characterized by a unique set of genes and functional properties. This functional divergence underscores the complexity of AT1 cell roles in maintaining pulmonary homeostasis and responding to various physiological and pathological stimuli.

### 3.2. AT1 Cells from Aged Human Lungs Showed Increased Cellular Senescence

**Figure 2.**
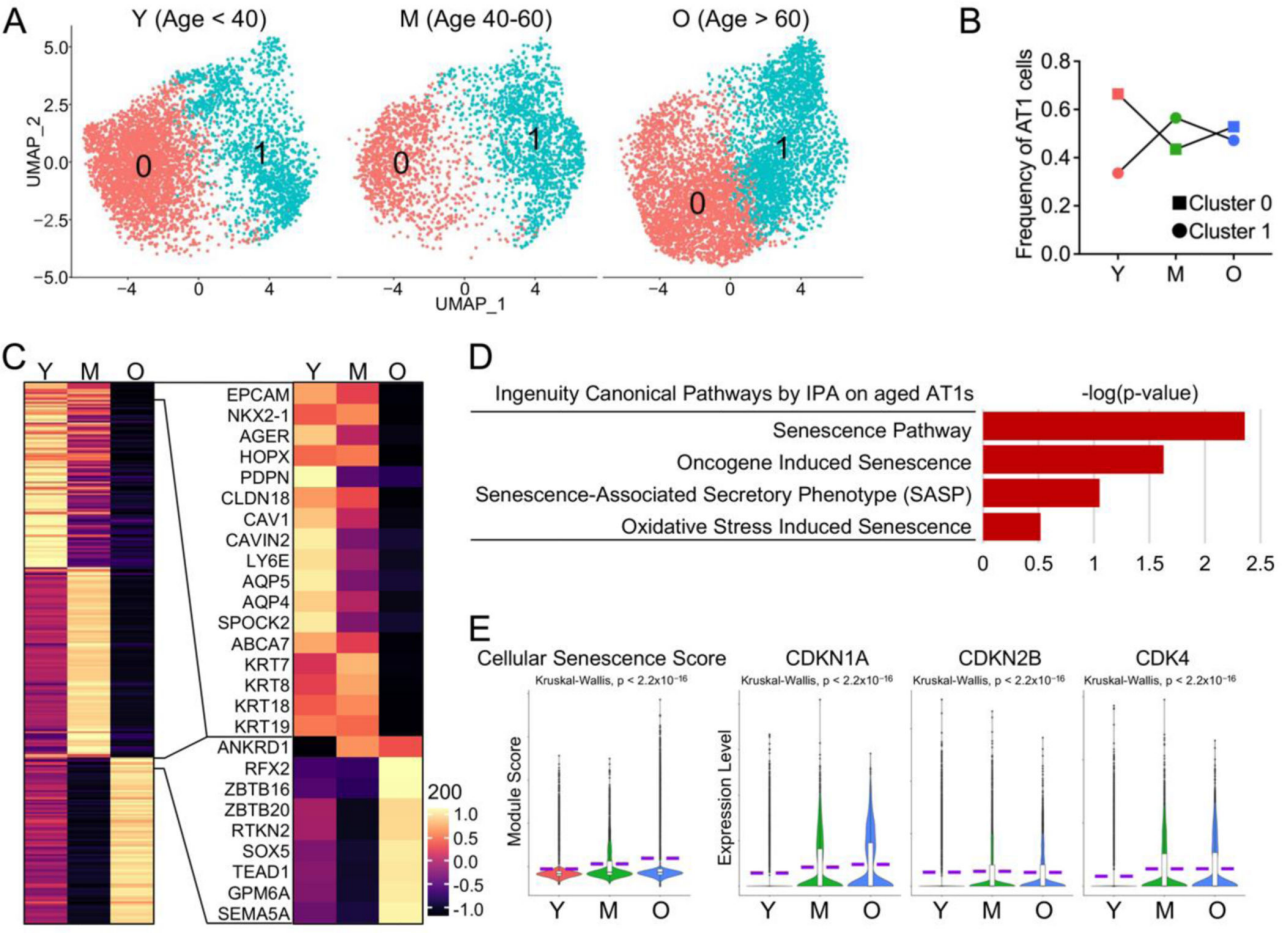
Increased cellular senescence in aged human AT1 cells. (**A**) UMAP visualization of AT1 clusters from lungs of young (Y, Age < 40), middle-aged (M, Age 40–60), aged (old) donors (O, Age > 60). (**B**) Frequency of AT1s in each sub-cluster from lungs of young, middle-aged, and aged (old) donors. (**C**) Heatmaps displayed the top 100 (left) and representative (right) genes differentially expressed in AT1s at different age states. (**D**) Cellular senescence-related pathways from IPA analysis in aged AT1s. (**E**) Violin plots showed the cellular senescence score and expression of representative senescence genes in AT1s of different age stages. Y, young (Age < 40), M, middle-aged (Age 40–60), O, aged (old) donors (Age > 60).

Aging has been suggested to lead to a gradual decline in lung function in healthy individuals, characterized by structural changes that hinder gas exchange, as well as immunologic changes that increase susceptibility to infections [6]. We propose that age-related compromises in human AT1 cells may contribute to the changes in lung function observed in healthy elderly individuals. To confirm this, we separated the AT1 cells into three groups by age as described previously [8]: young, <40 year old, middle-aged, 40–60 year old, and old (aged) >60 year old (Figure 2A). We quantified the frequencies of AT1 cells in each sub-cluster across different age stages and found that the frequency of Cluster 0 dropped dramatically with aging, while that of Cluster 1 increased (Figure 2B). To systematically characterize the profiles of AT1 cells across different age stages, we conducted differential expression analysis and identified distinct gene expression patterns in AT1 cells corresponding to each age stage (Figure 2C, left panel). Many of the highly expressed genes in young AT1 cells are related to the cellular structural integrity of epithelial cells, while many of those expressed in aged AT1 cells are transcriptional factor genes and their functions are yet to be determined (Figure 2C, right panel).

Cellular senescence, as one of the major common hallmarks of human aging [31], has been suggested to be involved in several age-related lung diseases [6]. To study the cellular senescence of AT1 cells, we ran IPA analyses and identified several cellular senescence related pathways in AT1 cells from aged subjects (Figure 2D). As a verification, we defined a cellular senescence score with the selected genes that are detectable in human alveolar epithelial cells from CSGene database (503 genes) [8,16,17] and found a significantly elevating cellular senescence score in AT1 cells with aging (Figure 2E). The upregulated expression levels of representative senescence genes, such as *CDKN1A* (P21), *CDKN2B* (P15), and *CDK4*, further confirmed the increased cellular senescence in aged AT1 cells (Figure 2E).

### 3.3. Decreased Cell Apoptosis in Aged AT1 Cells

**Figure 3.**
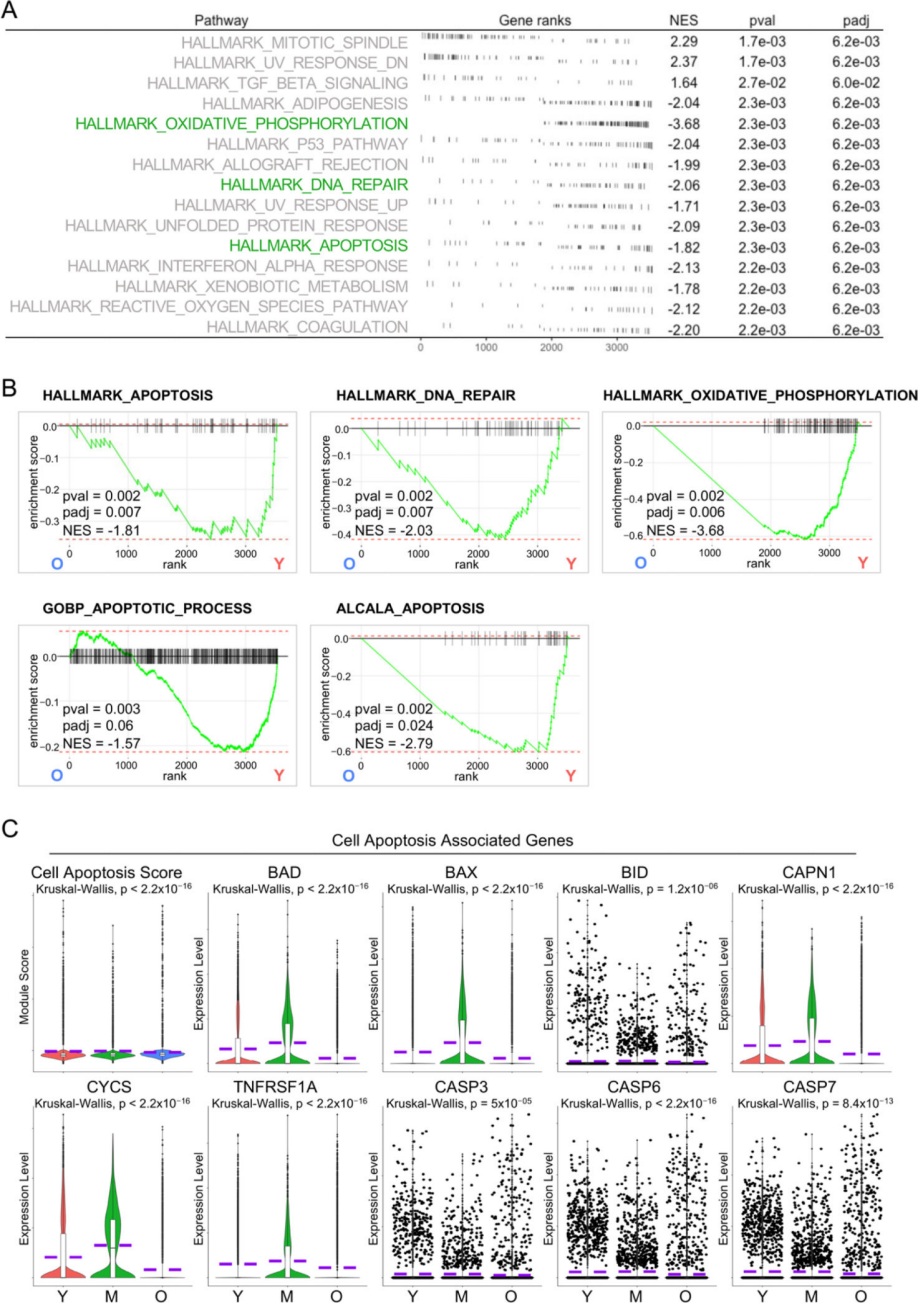
Reduced cell apoptosis in aged human AT1 cells. (**A**) Fast Gene Set Enrichment Analysis (GSEA) analysis revealed the top hallmark-pathways in AT1 cells from aged (O) vs young (Y) donors. (**B**) Enrichment plots showed the decreased apoptosis related pathways in aged AT1 cells by FGSEA analysis from HALLMARK, C2, and C5 Molecular Signatures Databases. (**C**) Violin plots showed the cell apoptosis score and the expression of representative cell apoptosis associated genes in AT1s of different age stages. Y, young (Age < 40), M, middle-aged (Age 40–60), O, aged (old) donors (Age > 60).

AT1 cells are often considered terminally differentiated cells in the adult lung although some studies believe that they retain cellular plasticity [32,33]. However, the consistency is that the turnover of AT1 cells is tightly regulated to maintain lung homeostasis. Programmed cell death, including apoptosis, is considered a natural mechanism regulating the turnover of AT1 cells and facilitating tissue remodeling processes and it is typically balanced by the differentiation of AT2 progenitor cells to replace the lost cells [34,35]. To further study the age-associated change in AT1 cells more systemically, we employed another pathway analysis method, fast gene set enrichment analysis (GSEA). Surprisingly, we identified several cell apoptosis related pathways downregulated in aged AT1 cells by analysis of Hallmark gene sets (Figure 3A,B). This finding was further corroborated by additional analyses on C2 curated gene sets and C5 ontology gene sets (Figure 3B). To validate the downregulated cell apoptosis in aged AT1 cells, we accessed the 81 protein-coding genes participating in the apoptosis pathway from the KEGG Pathways dataset and defined the Cell Apoptosis Score. Consistent with our GSEA analysis, the aged AT1 cells exhibited a significantly lower Cell Apoptosis Score and decreased transcription levels of representative cell apoptosis genes (Figure 3C). These data revealed insufficient AT1 cell apoptosis, indicating impaired turnover of AT1 cells and accumulation of damaged or senescent cells, which may lead to compromised lung tissue homeostasis and functionality in aging human lungs.

### 3.4. Decreased Epithelial Identities in AT1 Cells from Aged Subjects

**Figure 4.**
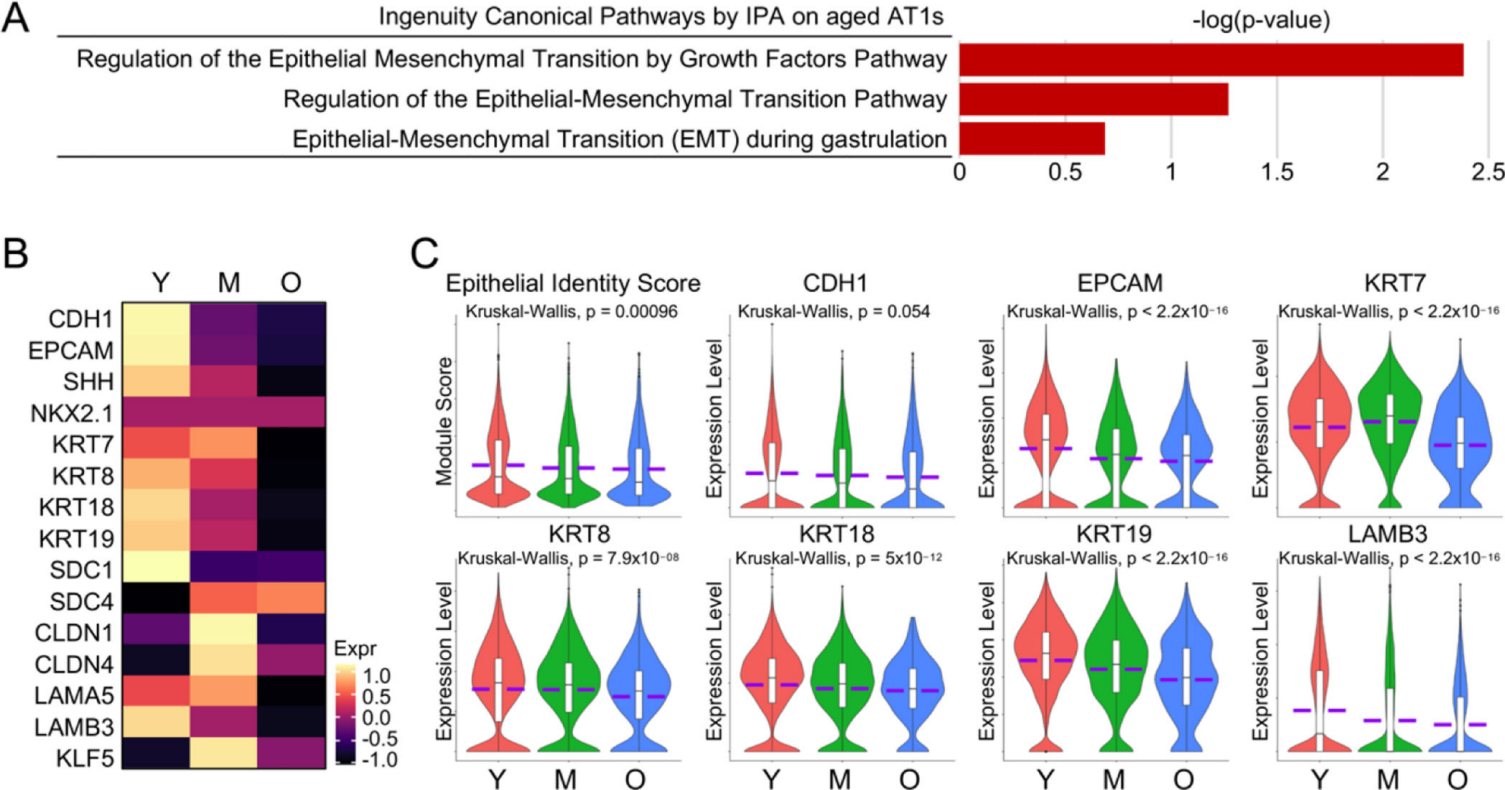
Reduced epithelial identities in aged human AT1 cells. (**A**) Epithelial-Mesenchymal Transition (EMT)-related pathways identified by IPA analysis on aged AT1s. (**B**,**C**) Heatmap (**B**) and violin plots (**C**) showed the Epithelial Identity Score and expression of representative epithelial identity genes in AT1s of different age stages. Y, young (Age < 40), M, middle-aged (Age 40–60), O, aged (old) donors (Age > 60).

Amidst the activated pathways identified by IPA analysis on aged AT1 cells, several intriguingly appeared to be associated with Epithelial-Mesenchymal Transition (EMT) (Figure 4A). Although we did not observe “real” EMT in aged AT1 cells, we did notice a consistent decrease in the transcription levels of common lung epithelial genes (Figure 4B). We then established an Epithelial Identify Score with the common lung epithelial genes and found that the Epithelial Identify Score, along with the representative epithelial cell marker genes, was significantly downregulated with the aging of human AT1 cells (Figure 4C). These observations, similar to the characterizations of aged AT2 cells as we reported recently [8], indicate a deficiency in epithelial features in AT1 cells from aged human lungs.

### 3.5. Dysregulated Cell Junctional Complexes in Aged Human AT1 Cells

**Figure 5.**
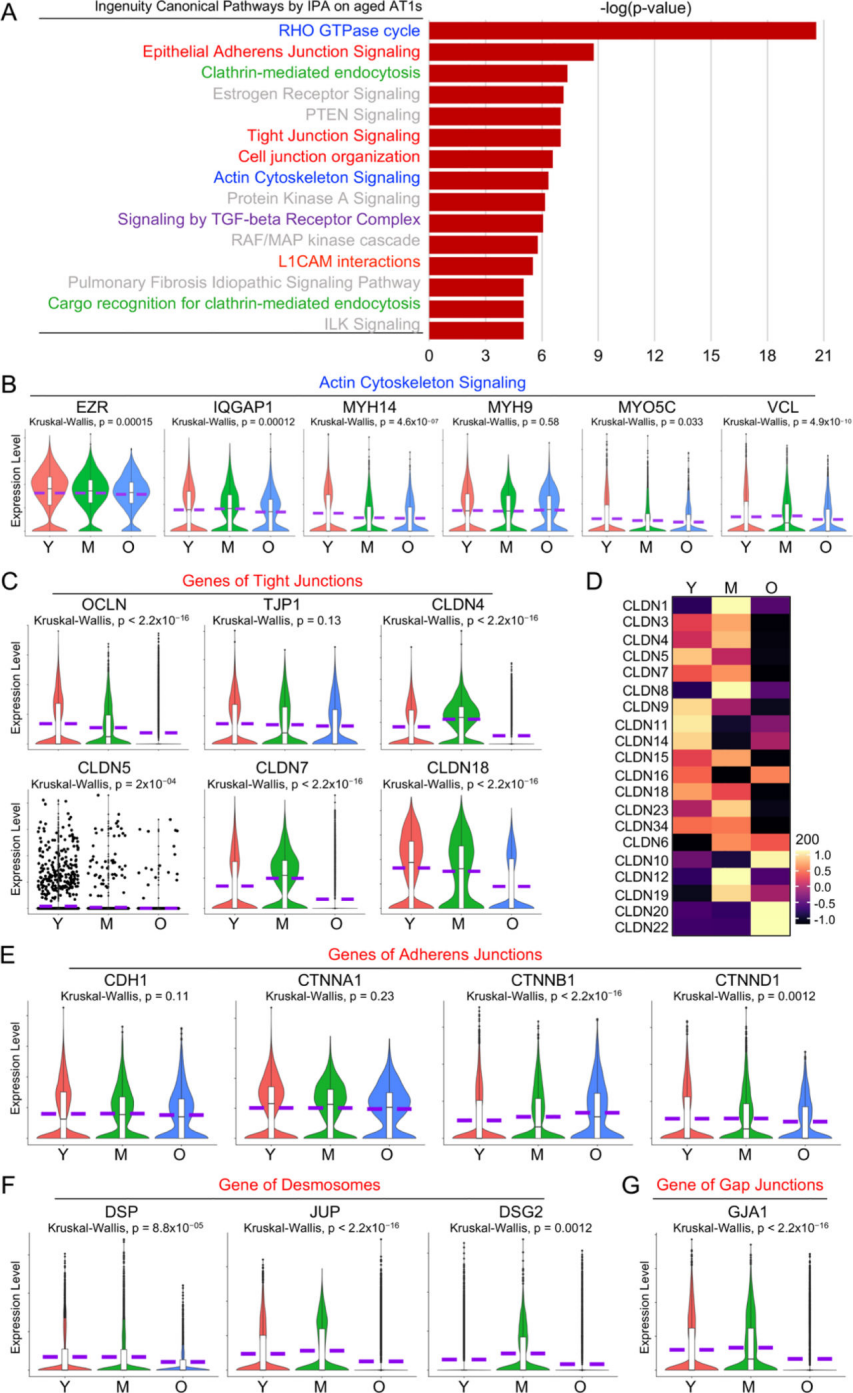
Dysregulated cell junctional complexes in aged human AT1 cells. (**A**) Top dysregulated Ingenuity Canonical Pathways by IPA analysis on aged AT1s were listed with interested pathways color-highlighted. (**B**) Representative genes of actin cytoskeleton signaling were visualized by violin plots. (**C**) Violin plots depicted the transcription levels of major tight junction components in AT1s of different age stages. (**D**) Heatmap displayed the expression levels of Claudin genes. (**E**–**G**) Gene transcriptional levels of key components in adherens junctions (**E**), desmosomes (**F**), and gap junctions (**G**) were illustrated by violin plots in AT1s of different age stages. Y, young (Age < 40), M, middle-aged (Age 40–60), O, aged (old) donors (Age > 60).

As one of the major features of epithelial cells, the intercellular junctions of alveolar epithelial cells are critical to form adhesive forces that connect neighboring cells and separate the external environment from the subepithelial tissue and conduct intercellular communications [36]. Among the top 15 dysregulated pathways identified by IPA analysis in aged AT1 cells, many are related to cell junction signaling (Figure 5A, red highlighted) and actin cytoskeleton signaling (Figure 5A, blue highlighted).

There are three major types of cell junctions: tight junctions, adherent junctions and desmosomes, and gap junctions and almost all require association with the actin cytoskeleton. To verify the dysregulated actin cytoskeleton and cell junction signaling, we then examined the transcriptional levels of the representative actin cytoskeleton genes and the major components of each cell junction complex in AT1 cells from different stages (Figure 5B–G). Many of the actin cytoskeleton component or intermediate genes were downregulated in the aged AT1 cells (Figure 5B). Tight junctions consist of transmembrane proteins (Claudins/*CLDN*s, Occludin/*OCLN*) and adaptor proteins (ZO-1/*TJP1* and ZO-2/*TJP2*) that link to the underlying actin cytoskeleton [37–39]. In aged AT1 cells, both *OCLN* and *TJP1*, along with most of the claudin genes, were significantly downregulated (Figure 5C,D). Adherens junctions are primarily composed of the transmembrane protein E-cadherin (encoded by *CDH1* gene) and adaptor catenin proteins [39]. Desmosomes are intercellular junctions requiring a protein complex formed by several major desmosomal proteins, such as desmogleins (*DSGs*), desmocollins (*DSCs*), junction plakoglobin (*JUP*), plakophilins (*PKP*), and desmoplakin (*DSP*) [40]. Many genes encoding the components of adherens junctions (Figure 5E) and desmosomes (Figure 5F) were also decreased in aged AT1 cells. Connexins are the major proteins of gap junctions [41]. One representative Connexins, Connexin-43/CX43 encoded by *GJA1* gene, which is detectable in human AT1 cells, was also dramatically downregulated in aged AT1 cells (Figure 5G). Age-related deficiency in cell junctions in AT1 cells can compromise lung integrity, disrupt efficient gas exchange, and increase susceptibility to respiratory diseases in elderly populations.

### 3.6. Dysregulated Cell Endocytosis in Aged AT1 Cells

**Figure 6.**
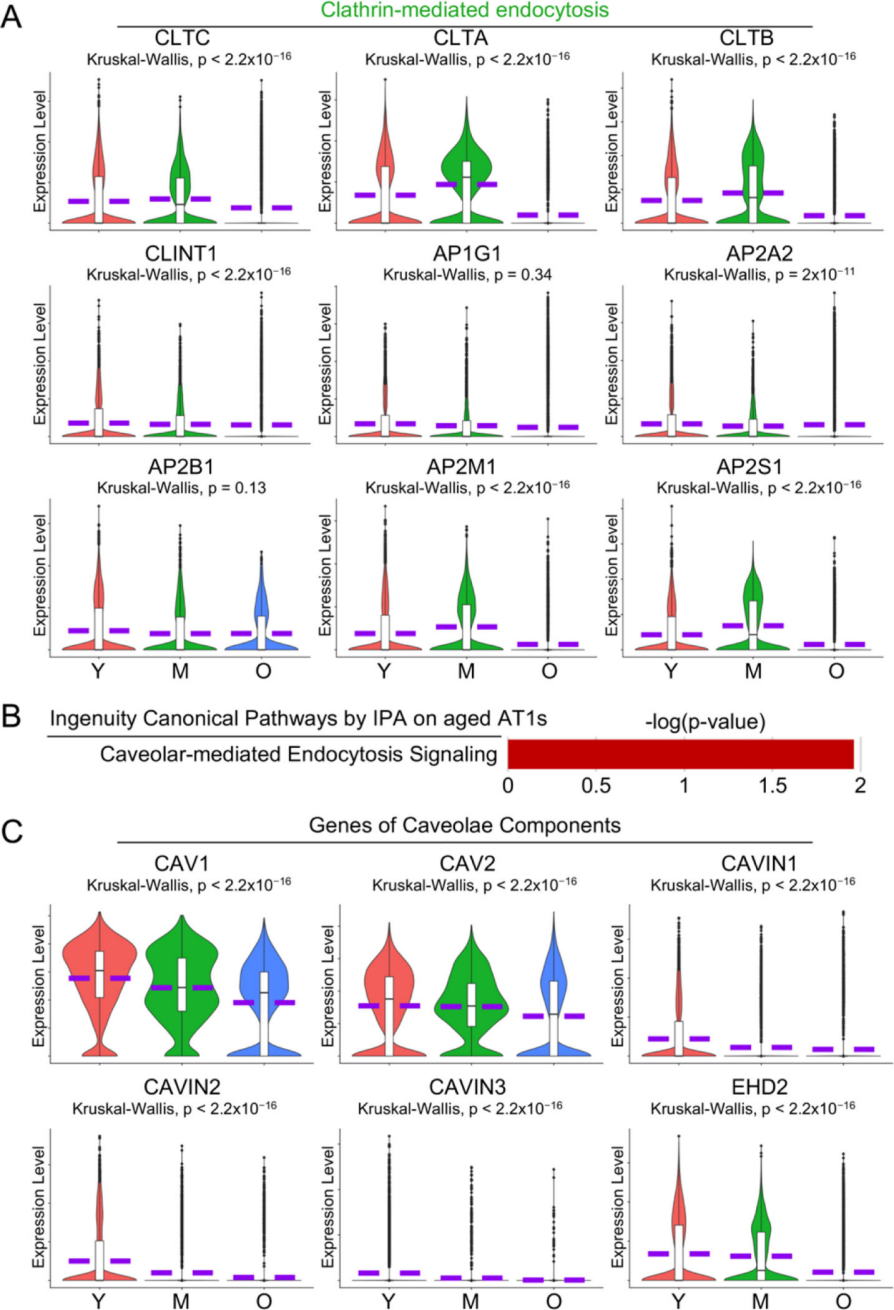
Impaired cell endocytosis in aged human AT1 cells. (**A**) Dysregulated Clathrin-mediated endocytosis was visualized by IPA analysis in Figure 3A, and violin plots showed the expression levels of the major components of Clathrin-mediated endocytosis. (**B**) IPA analysis suggested dysregulated Caveolar-mediated Endocytosis Signaling in aged AT1 cells. (**C**) Violin plots displayed the expression levels of the major components involved in caveolar-mediated endocytosis signaling. Y, young (Age < 40), M, middle-aged (Age 40–60), O, aged (old) donors (Age > 60).

Endocytosis in AT1 cells plays a crucial role in the uptake of extracellular materials, regulation of membrane protein turnover, and maintenance of cellular homeostasis, which are essential for efficient gas exchange and lung function [42,43]. IPA analysis on aged AT1 cells revealed several cell endocytosis-related pathways including Clathrin- (Figure 5A, green highlighted) and Caveolar-mediated endocytosis (Figure 6B). To validate these findings, we examined the transcriptional levels of the key components involved in clathrin-coated vesicle and caveolae, the major organelles mediating Clathrin- or Caveolar-mediated endocytosis, and found that most of these genes were significantly downregulated in aged AT1 cells (Figure 6A,C).

### 3.7. Compromised Pulmonary Matrisome Controlled by TGF-β in Aged AT1 Cells

**Figure 7.**
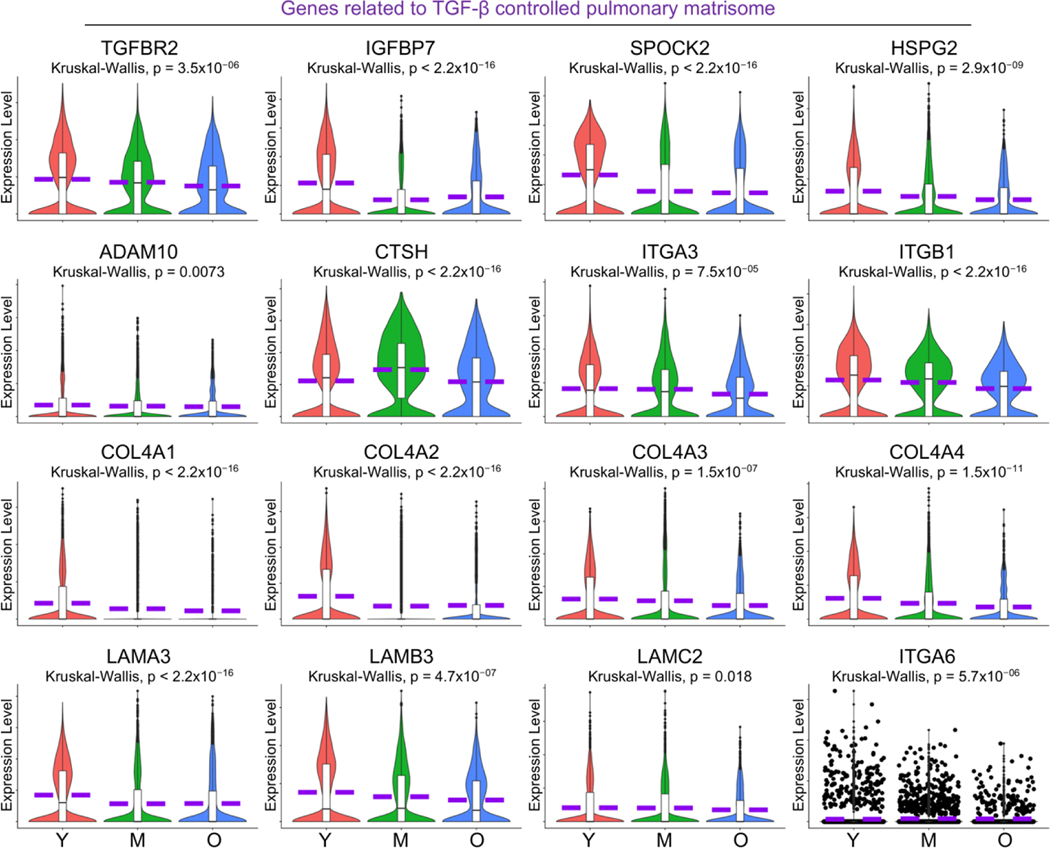
Deficient alveolar matrisome gene transcription by aged human AT1 cells. Reduced transcription level of *TGFBR2*, as well as genes related to ECM-related matrisome and Laminin-332 proteins, by aged human AT1 cells was visualized by violin plots. Y, young (Age < 40), M, middle-aged (Age 40–60), O, aged (old) donors (Age > 60).

The extracellular matrix (ECM) in the lungs is a unique structure that facilitates cell adhesion, migration, and differentiation, regulates tissue repair and remodeling processes, and ensures proper lung architecture and function [44]. A recent study demonstrates that numerous genes associated with constituents and regulatory components of the pulmonary ECM, collectively termed the pulmonary matrisome, are expressed in AT1 cells, and are regulated by TGF-β signaling through its receptor TGFBR2 in AT1 cells [45]. Here in the aged human lungs, we observed dysregulated signaling by TGF-β receptor complex by IPA analysis (Figure 5A, purple highlighted) and a deficient transcription level of *TGFBR2* (Figure 7) in aged AT1 cells. As expected, the expression levels of several pulmonary matrisome proteins, including glycoproteins (*IGFBP7*), proteoglycans (*SPOCK2* and *HSPG2*), ECM regulators (*ADAM10* and *CTSH*), and integrins (*ITGA3* and *ITGB1*) were significantly downregulated in AT1 cells with aging (Figure 7). Basement membrane provides essential support for AT1 cells by serving as a structural foundation that ensures their stability and proper alignment. Components of the basement membrane including the collagen IV subtypes (*COL4A1*, *COL4A3*, and *COL4A4*) and laminin-332 constituents (*LAMA3*, *LAMB3*, and *ITGA6*), are contributed by AT1 cells, but their transcriptional levels were markedly decreased in aged AT1 cells (Figure 7). Collectively, these data suggest an age-associated decline in the formation of the pulmonary matrisome by AT1 cells in aged human lungs.

### 3.8. Elevated Cell Inflammation in Aged AT1 Cells

**Figure 8.**
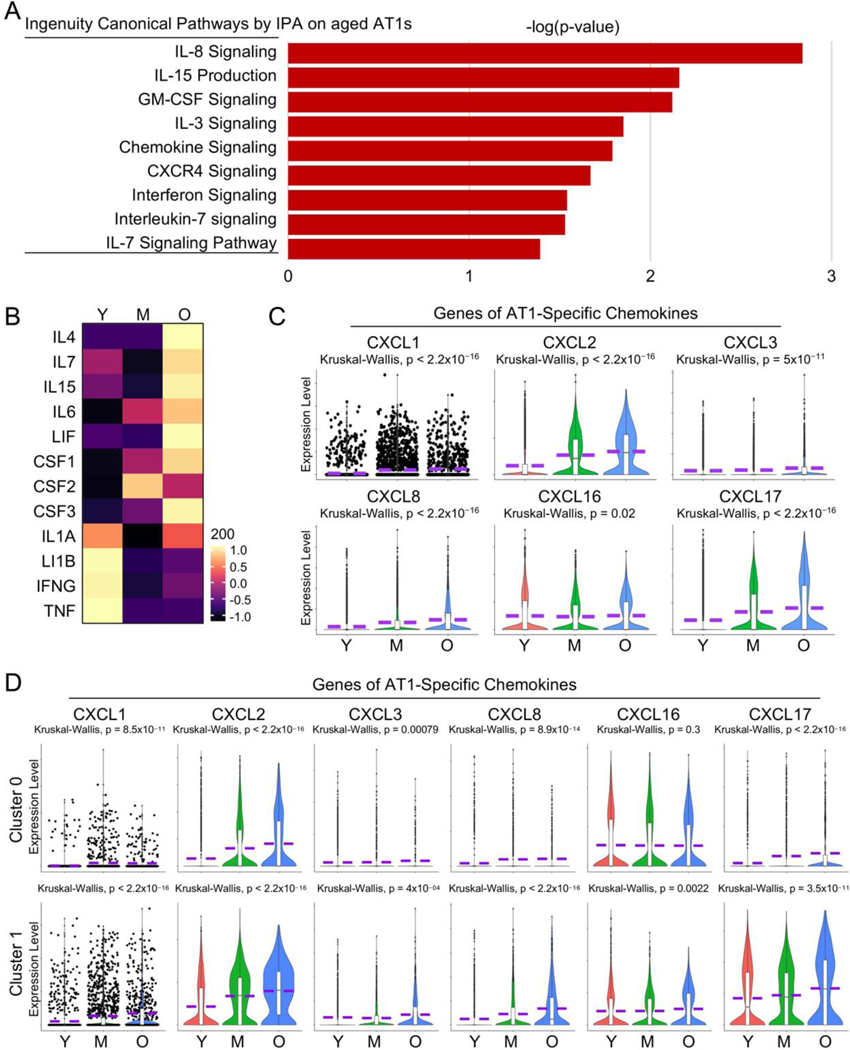
Increased cytokine signaling in aged human AT1 cells. (**A**) IPA analysis revealed several elevated cytokine and chemokine related signaling pathways in aged human AT1 cells. (**B**) Heatmap showed the expression of representative cytokine genes by AT1 cells of different age stages. (**C**) Violin plots illustrated elevated expression levels of AT1-cell specific chemokine genes. (**D**) Relative transcriptional levels of AT1-cell specific chemokine genes in AT1 cells of cluster 0 and 1 from different age stages. Y, young (Age < 40), M, middle-aged (Age 40–60), O, aged (old) donors (Age > 60).

Although covering a significant portion of the lung surface area exposed to the external environment, the potential contribution of AT1 cells to pulmonary immunity is not well understood [46]. Here our pathway analysis revealed several pathways associated with cell inflammation that were heightened in aged AT1 cells (Figure 8A). As more direct evidence shows, many cytokine genes, despite their overall low transcriptional levels, were upregulated in aged AT1 cells (Figure 8B). Meanwhile, the chemokine genes that could be detected in human AT1 cells were consistently up-regulated in aged AT1 cells (Figure 8C). Further analyses of separated AT1 subclusters indicated that both Cluster 0 and 1 contributed to the expression of these chemokine genes, although the overall transcription levels of these genes were much higher in Cluster 1 compared to Cluster 0 (Figure 8D). While the role of AT1 cells in pulmonary immunity has been rarely reported, the elevated cytokine gene expression by the aged AT1 cells suggests a newly identified function of AT1 cells in triggering innate immune responses in the human lung.

## 4. Discussion

Alveoli serve as the primary sites for blood-gas exchange within the human lung and alveolar epithelium, comprising two main cell types, alveolar type I cells (AT1) and alveolar type II cells (AT2), play a crucial role in this exchange process [44]. AT2 cells function as stem cells, aiding in the repair of injured alveolar epithelium [47]. AT1 cells are specialized epithelial cells that cover approximately 95% of the alveolar surface area in the lungs. Their distinctive squamous shape, along with their thin and extended surfaces, allows them to closely align with lung microvascular endothelial cells, thus facilitating the interface for efficient blood-gas exchange [1,48]. As individuals age, cells within the respiratory epithelium display both quantitative and qualitative differences and specific alterations associated with aging have been observed in most of the epithelial cell types in the both airway and alveolar regions [6]. While recent research has provided insights into age-related changes in lung structure and function, the distinct alterations occurring within AT1 cells have not been extensively characterized. Understanding how aging affects AT1 cells is crucial for elucidating the mechanisms underlying age-related lung dysfunction and may lead to the development of targeted therapeutic interventions to preserve respiratory health in the elderly population.

In the present study, we revisited a previously established single-cell transcriptomics atlas of healthy human lungs to delve into the gene expression dynamics of AT1 cells across different age groups. Our findings unveiled distinct gene expression profiles associated with aging in AT1 cells. Functional pathway analysis highlighted significant alterations induced by aging, including increased cellular senescence, diminished epithelial identities, and shifts in gene expression related to crucial cellular processes such as cell junctions, extracellular matrix dynamics, chemokine signaling, and apoptosis. These age-related changes have the potential to disrupt lung function, elevate susceptibility to respiratory ailments, and compromise overall respiratory health in the elderly population.

Cellular senescence, characterized by a permanent growth arrest, is recognized as one of the hallmarks of aging in human tissues [31]. Senescent cells accumulate with age and are thought to play a significant role in age-related tissue dysfunction [31]. Interestingly, senescent cells often exhibit resistance to apoptosis, a process of programmed cell death crucial for removing damaged or dysfunctional cells from tissues [49,50]. This phenomenon might explain the observed increase in cellular senescence in AT1 cells from aged human lungs, accompanied by a concurrent downregulation of cell apoptosis. The interplay between these two processes highlights the intricate mechanisms governing cellular homeostasis and aging in lung tissue, though further investigations are needed for validation. The imbalance of these cellular processes in alveolar epithelial cells is likely contributing to the development of age-associated lung diseases such as Idiopathic Pulmonary Fibrosis (IPF), Chronic Obstructive Pulmonary Disease (COPD), and lung cancer, although direct evidence is still lacking.

Another hallmark of aging in human tissues is altered intercellular communication [31]. One of the primary features of epithelial cells is the presence of cell junctions, which are considered crucial mechanisms for intercellular communication of epithelial cells [39]. Thus, impaired gene expression of cell junction complexes in aged AT1 cells may indicate not only compromised epithelial integrity and barrier, but also disrupted intercellular communications of AT1 cells with their neighboring cells or the extracellular matrix in aged human lungs. Endocytosis is indeed another mechanism of cell communication, which plays a crucial role in various cellular processes that facilitate intercellular communication and signal transduction [51]. Dysregulated endocytosis in aged AT1 cells, including clathrin- and caveolae-mediated endocytosis, underscores the compromised cell communication in these cells. We hypothesize that other endocytosis pathways specific to epithelial cells may also be affected by aging, although they were not explored in the current study. Another recently identified function of AT1 cells is their role in maintaining alveolar matrisome through ECM secretion [45]. These components, along with other ECM elements such as the basement membrane, play a crucial role in preserving alveolar integrity. Our recent analyses using the same integrated dataset have shown a decreased transcriptional level of ECM genes, including basement membrane genes, in mesenchymal cells from aged human lungs [8]. These findings collectively suggest a compromised signaling interplay between AT1 cells and their neighboring cells, leading to a deficit in alveolar integrity and intercellular communication in aged human lungs. Pulmonary diseases such as IPF, COPD, pneumonia, and lung cancer are more prevalent with increasing age and are commonly associated with disruptions in alveolar integrity and structure. Age-related molecular and cellular changes in AT1 cells may contribute to the pathogenesis of these pulmonary diseases, though more direct evidence is needed to confirm this involvement.

AT2 cells, as the progenitor cells, have also been reported to execute various innate immunologic activities and participate in lung adaptive immune responses as well [8,52–54], but the role of AT1 cells in pulmonary immunity has been rarely studied. Here, we reported an increased transcription of cytokine genes in aged human AT1 cells. Although the precise functions of these genes remain unclear, we propose that AT1 cells participate in pulmonary immunity via the secretion of these chemokines and future studies will be actively pursued.

## 5. Conclusions

In summary, this study revealed a heterogeneity within AT1 cells, with distinct subpopulations identified, with a recently generated single-cell transcriptomics atlas of healthy human lungs. Aged AT1 cells exhibited increased cellular senescence, elevated chemokine gene expression, and resistance to apoptosis, along with a deficiency in epithelial identities and altered gene expression related to cell junctions, pulmonary matrisome, and endocytosis. These findings underscore the impact of aging on AT1 cells and highlight implications for respiratory health. Understanding these mechanisms could inform targeted therapies to preserve lung function and improve respiratory health in the elderly.
